# Misuse of the Term Retrospective Cohort. Comment on Alsayouf et al. Atomoxetine Treatment of Attention Deficit/Hyperactivity Disorder Symptoms in 3–6-Year-Old Children with Autism Spectrum Disorder: A Retrospective Cohort Study. *Children* 2024, *11*, 163

**DOI:** 10.3390/children11121539

**Published:** 2024-12-19

**Authors:** Mehran Zarghami

**Affiliations:** 1 Psychiatry and Behavioral Sciences Research Center, Addiction Institute, Mazandaran University of Medical Sciences, Sari 4843185774, Mazandaran, Iran; mzarghami@mazums.ac.ir or mehran.zarghami@gmail.com; 2 Department of Psychiatry, Faculty of Medicine, Mazandaran University of Medical Sciences, Sari 4843185774, Mazandaran, Iran

## Abstract

Recently, an article entitled “Atomoxetine Treatment of Attention Deficit/Hyperactivity Disorder Symptoms in 3–6-Year-Old Children with Autism Spectrum Disorder: A Retrospective Cohort Study” was published in the journal *Children*. In this letter, the methodology of the mentioned study is discussed.

I read with interest the recently published article in the “*Children*” by Alsayouf et al., entitled “Atomoxetine Treatment of Attention Deficit/Hyperactivity Disorder Symptoms in 3–6-Year-Old Children with Autism Spectrum Disorder: A Retrospective Cohort Study” [1], which deals with an important issue in the field of child and adolescent psychiatry. The esteemed authors had reviewed the charts of several patients who were suffering from attention deficit/hyperactivity disorder (ADHD) comorbid with autism spectrum disorder (ASD) and who were previously treated with atomoxetine. They investigated the outcomes of this treatment, which included examining the safety profile and effectiveness of atomoxetine (that were already available at the time of the study). The authors indicated the type of study as a retrospective cohort study.

The point that I am presenting about this article is related to research methodology. Unfortunately, when researchers describe their study design, in many cases, the term “cohort”, especially “retrospective cohort”, is misused [2]. In a cohort study, participants initially do not have the outcome of interest. They are selected based on the exposure status of the person. Therefore, classically, there are the following two groups of participants at baseline: a group of study participants who have had exposure (defined as the exposed group) and others who have had no exposure (defined as the unexposed group). They are then followed over time to assess the occurrence of the outcome of interest. During this follow-up period, some exposed individuals achieve the outcome of interest; some unexposed people may also achieve the desired outcome [3,4].

In prospective cohort studies, all data are collected prospectively. In the above-mentioned study, the researchers defined the population to be included in the cohort. They then measured the exposure of interest (defined as exposure to atomoxetine treatment in their article). An important point to note is that the non-exposed people were not studied and only the outcomes of the exposed people were examined. Another important point is that in cohort studies, the researchers also collect information about confounding variables at the baseline and during follow-up [4]. But Alsayouf et al. did not examine the confounding variables that might have influenced the outcomes.

It should be noted that in retrospective or historical cohort studies (that is, the type of study which is mentioned in the article being discussed), the exposure and outcomes have happened in the past, and the data are collected from various registers. Although the outcomes have already happened, the basic research structure is essentially the same [2,4,5].

We should pay attention to the fact that the authors of the article did not perform an intervention but described an intervention that had already been carried out. Therefore, referring to this as a “retrospective cohort study” is not entirely accurate, although it may not be strictly incorrect if loosely defined. According to the above explanation, I think it would be better to say that Alsayouf et al.’s article is a “retrospective observational”, “retrospective descriptive”, “case series” (also known as “clinical series”) or simply a “chart review”. However, it is appropriate to add the adverb “non-interventional” to the type of study; as in Austria, studies involving the collection of retrospective medical records are classified as “Nicht-interventionellen Studie” [6]. This study is a type of medical study that describes the characteristics and outcomes of a group of people (without a control group) who are exposed to a risk factor or experience exposure or intervention over a period of time. Data are gathered retrospectively or prospectively, and no randomization is performed. As Alsayouf et al. have described in their article, in these studies, the goal is to describe the population and their outcomes, not to compare the risks between different groups. Therefore, case series differ from cohort studies because the latter compares the risks between two groups (exposed and unexposed) and allows for the absolute risk of a certain outcome to occur in the exposed group, as well as the comparison of relative risk with the unexposed group [7]. This is something that was not performed in the study by Alsayouf and colleagues. Of course, the criticism of the methodology stated in their article does not diminish the value of their study.
